# Are trajectories of social isolation from childhood to mid-adulthood associated with adult depression or suicide outcomes

**DOI:** 10.1007/s00127-022-02389-6

**Published:** 2022-12-02

**Authors:** Roy Lay-Yee, Timothy Matthews, Terrie Moffitt, Richie Poulton, Avshalom Caspi, Barry Milne

**Affiliations:** 1 Centre of Methods and Policy Application in the Social Sciences, School of Social Sciences, Faculty of Arts, University of Auckland, Auckland, New Zealand; 2 Department of Social Genetic and Developmental Psychiatry, Institute of Psychiatry, King’s College London, London, UK; 3 Department of Psychology and Neuroscience, Duke University, Durham, NC USA; 4 Department of Psychology, University of Otago, Dunedin, New Zealand; 5 Department of Statistics, Faculty of Science, University of Auckland, Auckland, New Zealand

**Keywords:** Social isolation, Life course, Child, Adult, Depression, Suicide, Mental health

## Abstract

**Purpose:**

Social isolation has been shown to have negative effects on mental health outcomes though little is known about trajectories across the life course. We examined the relationship between trajectory groups and selected mental health outcomes in mid-adulthood.

**Methods:**

We previously created a typology of social isolation based on onset during the life course and persistence into adulthood, using group-based trajectory analysis of longitudinal data from a New Zealand birth cohort. The typology comprises four groups: ‘never-isolated’, ‘adult-only’, ‘child-only’, and ‘persistent (child–adult) isolation’. We undertook logistic regression analyses of three mental health outcomes with trajectory group as the predictor, adjusting for sex and a range of familial and child-behavioural factors.

**Results:**

Lifetime suicide attempt, and depression and suicide ideation in mid-adulthood were each associated with adult-only but not child-only social isolation. Depression in mid-adulthood was also associated with persistent child–adult social isolation.

**Conclusion:**

Although our findings are associational and not causal, they indicate that interrupting persistent social isolation may help to prevent adult depression whereas halting adult social isolation may ameliorate both depression and suicide outcomes.

## Introduction

There has been growing concern—particularly in western countries—that social isolation poses a serious threat to public health and warrants high-priority intervention [1, 2]. This has only been exacerbated by the social strictures implemented to combat the COVID-19 pandemic [3]. Humans need social connection—thought to confer the individual with psycho-social and material resources—to maintain good health and well-being [4, 5]. For example, higher stocks of social capital have been linked to good mental health [6–8]. In contrast, the degree to which an individual is isolated—that is mostly alone and lacking any or having little social contact with others—limits opportunities for needs to be met [9–11].

Social isolation is not an uncommon occurrence though its prevalence is linked to particular risk factors, especially aspects of social disadvantage [12]. For example, in children, family environment and child socio-emotional factors have been associated with social isolation [13–15]. Social isolation can affect individuals at any age as they experience the challenges of each life stage, it may have an earlier or later onset in life, and it may be transient or persistent in duration. It has been found that social isolation is more strongly correlated with poor health at younger than at older ages [16], and that the prevalence of depressive symptoms [17] and of suicidal ideation or behaviour [18] both appear to be greater in adolescence than in adulthood. To date, most studies of social isolation have been cross-sectional and focussed on older people [19]. Longitudinal investigations of social isolation from childhood into adulthood are important to understand the development of social isolation and its relationship to negative outcomes such as poor mental health [20, 21]. A life-course perspective is beneficial in considering earlier influences and indicating possible points for intervention to reduce risk of harm in adulthood [22, 23].

There is evidence that poor peer relationships in childhood are associated with mental health problems [24–26], and that social isolation has negative consequences for the child’s social and emotional functioning [27–30]. More pointedly, it has been shown that social isolation in school-aged children is related to a range of mental health symptoms [13], and that socially isolated adolescents are at greater risk of long-term mental health problems [31] including depressive symptoms [32]. Further, social isolation occurring in childhood may have continuing adverse effects as children develop into their adult selves. Adults may suffer worse mental health outcomes as a result of earlier social isolation, for example, depression [33, 34], and suicide [35]. The relationships are not straightforward, e.g., lifetime adversities may mediate the relationship between childhood neglect or abuse—a risk factor for social isolation—and chronic or recurrent adult depression [36]. The long-term impacts of the onset and duration of social isolation have been seldom investigated [21] but have implications for policy and practice. For example, if childhood social isolation has negative impacts regardless of whether isolation persists into adulthood, this would argue for prevention strategies targeted towards childhood. Conversely, if only long-lasting social isolation is associated with negative consequences, then this would argue for prevention strategies which involve either waiting until adolescence (to see which individuals have long-lasting social isolation) or directing towards children with profiles suggestive of long-lasting social isolation [37].

This paper is focussed on addressing the gap in understanding of the dynamic relationship between social isolation and mental health over the life course. We have recently identified four distinct trajectory groups of social isolation (low, increasing, decreasing, and high), and have demonstrated that these have different risk factor profiles by family and child characteristics [38]. Here, we aim to investigate the degree to which these trajectory groups are related to a set of adult mental health outcomes that have been consistently associated with social isolation in cross-sectional studies, namely depression [39, 40] and suicide outcomes [41, 42]. Our research question is: Does membership of ‘trajectory’ groups influence adult depression, lifetime suicide attempt or recent suicide ideation? We hypothesize that patterns of social isolation in children and adults affect these adult mental health outcomes. We expect that adult mental health will be worse in groups that have experienced social isolation during their life course, and worst in the group with persistent social isolation.

## Methods

### Data sources

We used data from the Dunedin Multidisciplinary Health and Development Study (DMHDS), an ongoing longitudinal investigation of the health and behaviour of a complete birth cohort of consecutive births (*N* = 1037) over a 1-year period from April 1, 1972, to March 31, 1973, in Dunedin, New Zealand [43]. The DMHDS has been approved by the New Zealand Health and Disability Ethics Committee, and informed consent was obtained from participants. To date, assessments have been carried out at ages 3, 5, 7, 9, 11, 13, 15, 18, 21, 26, 32, 38, and 45. We focus on assessments of outcomes at age 38 which took place in 2010–2012 when 95% (*n* = 961) of living cohort members participated in the study, and at age 45 in 2017–2019 when 94% (*n* = 938) participated. The study samples include participants who had data collected on social isolation at ages 5–11 and 26–38, as well as on mental health conditions in both childhood and adulthood.

### Mental health outcome variables

The DMHDS mental health assessment using the Diagnostic Interview Schedule (DIS) [44] allows for diagnoses and symptom scores for depression, whether the study member has ever attempted suicide, and recent suicide ideation.

#### Depression

At ages 38 and 45 years, diagnoses of depression in the last 12 months were made according to DSM-IV criteria for Major Depressive Episode. A binary variable was derived to signify depression at age 38 or 45 years (yes/no).

#### Lifetime suicide attempt

At ages 38 and 45 years, participants were assessed as to whether they had ever attempted suicide. A binary variable was derived to signify suicide attempt by age 38 or 45 years (yes/no).

#### Recent suicide ideation (secondary outcome)

At ages 38 and 45 years, participants were assessed as to suicide ideation in the past year—“Did you think a lot about committing suicide?” A binary variable was derived to signify suicide ideation at age 38 or 45 years (yes/no).

### Social isolation exposure

Social isolation in childhood at ages 5, 7, 9, and 11, and again in adulthood at ages 26, 32, and 38, has been used to statistically derive four trajectory groups: never isolated (neither isolated as a child nor as an adult); child only (isolated as a child but not as an adult), adult onset (first became isolated as an adult), and child-and-adult (isolated both as a child and as an adult) [38]. Membership of these groups represents 30-year trajectories in change of social isolation status. *Child isolation* was assessed by a collection of measures [21, 33] whereby their parent and teacher completed the Rutter Child Scale [45], reporting on two items that measure peer problems: ‘tends to do things on his/her own; is rather solitary’ and ‘not much liked by other children’. At each age, scores on these two scale items (0 = doesn't apply, 1 = applies somewhat, 2 = certainly applies) were averaged across the two reporting sources (i.e., parent and teacher). *Adult isolation* was assessed using informant report whereby up to three informants whom the study member nominated as ‘knowing them well’ was mailed a questionnaire. At each age, over 90% of study members had reports from at least two informants and over 60% had reports from all three informants. At each age, scores on the item ‘seems lonely’ (0 = not a problem, 1 = bit of a problem, 2 = yes, a problem) were averaged across informants.

### Confounders

A number of family and child-behavioural factors have been found to be associated with social isolation trajectory group membership [38], and these may potentially confound associations between social isolation and mental health outcomes. In addition, sex (defined in binary terms: female/male), the child’s family socio-economic status, and previous mental health were used as confounders in analyses.

#### Family factors

i. A score for *family socio-economic status* was estimated as the average of the higher level of either parent using the Elley-Irving scale of occupational socio-economic status [46] which was assessed repeatedly at the study member’s birth and at ages 3, 5, 7, 9, 11, 13, and 15 years. Individual scores were grouped into low (16%), medium (63%), and high (21%) categories.
ii. A *teen-aged mother* was defined as being aged 18 or under and coded in a binary variable as ‘yes’ or ‘no’.
iii. We assessed whether the child had a *single parent* for at least one year up to age 11 as a binary variable coded ‘yes’ or ‘no’.
iv. We measured *change in residence* as the number of times that this occurred up to age 11.
v. An index of *maltreatment* was formed by combining measures of maternal rejection (age 3), harsh discipline (ages 7 and 9), disruptive caregiver changes from birth to age 11, exposure to physical abuse from birth to age 11, and exposure to sexual abuse from birth to age 11 [47].

#### Child-behavioural factors

i. *Self-control*: or the ability to regulate emotions and behavior—was assessed using a scale combining measures of lack of control (ages 3 and 5), impulsivity (ages 5, 7, 9, and 11), and hyperactivity (ages 5, 7, 9, and 11) [48]. The scale was split into quintiles for the purposes of analysis (quintile 1 = highest self-control; quintile 5 = lowest self-control).
ii. When a study member was 5, 7, 9, and 11 years old, their parent and teacher completed the Rutter Behavior Scales [45]. Scores on the *worried/fearful* scale—a measure of childhood internalizing symptoms—were averaged across rater and age and split into quintiles for analysis and interpretation (note, items relating to social isolation were not used in the construction of the worry/fearful scale).

#### Previous mental health

In addition, previous depression (major depressive episode) as an adolescent (at ages 11, 13, or 15 years) was also controlled for. It has been shown that having a mental health disorder at an earlier life stage is associated with having a disorder at a later stage [49]. In particular, higher levels of depression in childhood or adolescence are related to a higher risk of suffering from a mental disorder in adulthood [17, 50].

### Data analysis

In a previous paper, we used group-based trajectory modelling [51] to classify individuals exhibiting similar social isolation trajectories over the life course from childhood to adulthood (to age 38 years) [38]. The typology comprises four groups according to the onset and duration of isolation: never-isolated (*n* = 710, 71.6% of the cohort), child-only (*n* = 142, 14.3%), adult-only (*n* = 100, 10.1%), and child-and-adult (*n* = 40, 4.0%) [38]. Here, we employed that same typology of trajectory groups to examine its relationship to adult mental health outcomes—depression and recent suicide ideation at ages 38 or 45 years, and lifetime suicide attempt by ages 38 or 45. Firstly, we ascertained the distribution of each adult mental health measure among the predictor of interest (i.e., the trajectory groups) and across categories within potential confounders. Secondly, we undertook logistic regression modelling to ascertain whether binary depression, lifetime suicide attempt or recent suicide ideation, respectively, in adulthood were predicted by trajectory group membership while adjusting for family and child-related confounders previously shown to be important risk factors for social isolation—i.e., having a teenaged mother, having a single parent, changes in residence, maltreatment, self-control, and worry/fearfulness [38]—as well as sex of the individual, family socio-economic status, and adolescent depression. Analyses were carried out using Stata 16.0 [52].

## Results

In the conduct of the DMHDS—whose data were used for the analyses in this paper—great care has been taken to minimize missing data and attrition over the course of the study [43]. The cohort members were aged 38 when their data were used to estimate the social isolation trajectories, with little loss to follow-up due to dropout or mortality. At age 38, 95% of living cohort members participated in the study, and, at age 45, 94% participated. Moreover, there was no evidence that (i) those not assessed at a later time point (ages 26–38) were more likely to report social isolation at an earlier time point (ages 5–11); (ii) those who died by age 38 differed in their early isolation experience; nor (iii) those missing data for the three main outcomes differed in their early isolation experience.

### Bivariate associations

Overall, the prevalence of diagnosed depression (at ages 38 or 45) was 25.4%, of lifetime suicide attempt (by ages 38 or 45) was 13.6%, and of recent suicide ideation (at ages 38 or 45) was 6.2% (Table 1). Adult mental health outcomes for the four ‘trajectory’ groups are also shown in Table 1. Each of the three groups experiencing any degree of social isolation have higher levels of depression, lifetime suicide attempt, and recent suicide ideation, compared to the ‘never isolated’ group. Moreover, there was a gradient of prevalence ranging from that for the ‘never isolated’ group (lowest), through child-only and adult-only, to the persistent ‘child and adult’ group (highest) for each outcome (except recent suicide ideation where the adult-only group showed the highest proportion). In detail, the prevalence ranged: (i) for depression, from 19.6% (never-isolated group) to 57.1% (child-and-adult group); (ii) for lifetime suicide attempt, from 11.0% (never-isolated group) to 30.0% (child-and-adult group); and (iii) for recent suicide ideation, from 3.8% (never-isolated group) to 20.8% (adult-only group).

**Table 1:** Mental health outcomes at ages 38/45 years by ‘trajectory’ group, family–child factors, and previous mental health

Covariates	Depression at 38/45 *n/N* (% diagnosed)	Suicide attempt lifetime *n/N* (% attempt)	Suicide ideation at 38/45 *n/N* (% ideation)
	244/959 (25.4%)	138/1017 (13.6%)	59/958 (6.2%)
Trajectory group			
Never isolated	133/678 (19.6%)	78/710 (11.0%)	26/678 (3.8%)
Child only	39/133 (29.3%)	16/142 (11.3%)	8/133 (6.0%)
Adult only	48/97 (49.5%)	28/100 (28.0%)	20/96 (20.8%)
Child and adult	20/35 (57.1%)	12/40 (30.0%)	4/35 (11.4%)
Sex			
Female	140/478 (29.3%)	76/494 (15.4%)	33/478 (6.9%)
Male	104/481 (21.6%)	62/523 (11.9%)	26/480 (5.4%)
Family socio-economic status			
Low	62/193 (32.1%)	47/213 (22.1%)	19/193 (9.8%)
Medium	143/605 (23.6%)	76/637 (11.9%)	36/605 (6.0%)
High	38/156 (24.4%)	14/161 (8.7%)	4/155 (2.6%)
Teen-aged mother			
Yes	39/95 (41.1%)	22/99 (22.2%)	11/95 (11.6%)
No	204/860 (23.7%)	115/914 (12.6%)	48/859 (5.6%)
Single parent for at least a year, up to age 11			
Yes	43/130 (33.1%)	34/139 (24.5%)	11/130 (8.5%)
No	196/818 (24.0%)	100/865 (11.6%)	46/817 (5.6%)
Change in residence			
(Number) 0	55/284 (19.4%)	30/302 (9.9%)	10/284 (3.5%)
1	57/215 (26.5%)	31/229 (13.5%)	14/215 (6.5%)
2	32/150 (21.3%)	24/161 (14.9%)	7/149 (4.7%)
3+	100/310 (32.3%)	53/324 (16.4%)	28/310 (9.0%)
Maltreatment			
Probable/severe	113/346 (32.7%)	72/270 (19.5%)	30/345 (8.7%)
None	131/613 (21.4%)	66/647 (10.2%)	29/613 (4.7%)
Self-control			
Quintile 1	36/189 (19.1%)	17/197 (8.6%)	8/189 (4.2%)
Quintile 2	37/194 (19.1%)	19/205 (9.3%)	11/194 (5.7%)
Quintile 3	52/196 (26.5%)	25/206 (12.1%)	9/196 (4.6%)
Quintile 4	60/196 (30.6%)	32/206 (15.5%)	13/195 (6.7%)
Quintile 5 (low)	59/184 (32.1%)	45/203 (22.2%)	18/184 (9.8%)
Worry/fearfulness			
Quintile 1	21/134 (15.7%)	11/141 (7.8%)	1/134 (0.8%)
Quintile 2	48/215 (22.3%)	29/226 (12.8%)	14/215 (6.5%)
Quintile 3	40/176 (22.7%)	18/186 (9.7%)	13/176 (7.4%)
Quintile 4	63/214 (29.4%)	31/230 (13.5%)	21/214 (9.8%)
Quintile 5 (high)	71/212 (33.5%)	46/223 (20.6%)	10/211 (4.7%)
Depression, ages 11–15			
Yes	26/53 (49.1%)	26/58 (44.8%)	8/52 (15.4%)
No	207/871 (23.8%)	108/917 (11.8%)	47/871 (5.4%)

Family and child characteristics—as well as sex of the child and socio-economic status—were also associated with adult mental health outcomes (Table 1). Adults who were born to teen-aged mothers or raised by single mothers, or who, as children, experienced frequent changes in residence or were maltreated, had higher proportions with depression, lifetime suicide attempt, and recent suicide ideation. Adults who as children exhibited low self-control or tended to be worried or fearful, or who as adolescents suffered from depression, also showed higher levels of the three mental health outcomes.

### Logistic regression analyses

For adult depression, ‘trajectory’ group membership—belonging in any one of the isolated groups—was a risk factor for adult depression in unadjusted analysis (Table 2). After adjustment for confounders, the ‘adult-only’ group (OR 3.25, 95% CI 1.99–5.32, *p* < 0.001) and the ‘child-and-adult’ group (OR 4.20, 95% CI 1.84–9.61, *p* = 0.001) remained at significantly higher risk than the ‘never-isolated’ group, but this was no longer the case for the ‘child-only’ group.

**Table 2:** Relationship between ‘trajectory’ group and mental health outcomes at ages 38/45 years

Trajectory group	Depression at 38/45		Suicide attempt lifetime		Suicide ideation at 38/45	
	Odds ratio (95% CI), *p*		Odds ratio (95% CI), *p*		Odds ratio (95% CI), *p*	
	Raw^a^	Adjusted^b^	Raw^a^	Adjusted^b^	Raw^a^	Adjusted^b^
Never isolated	Ref^c^	Ref^c^	Ref^c^	Ref^c^	Ref^c^	Ref^c^
Child only	1.70 (1.12–2.58) *p* = 0.013*	1.21 (0.75–1.94) *p* = 0.439	1.03 (0.58–1.82) *p* = 0.922	0.51 (0.25–1.03) *p* = 0.059	1.60 (0.71–3.63) *p* = 0.255	1.46 (0.59–3.57) *p* = 0.410
Adult only	4.01 (2.58–6.24) *p* < 0.001*	3.25 (1.99–5.32) *p* < 0.001*	3.15 (1.92–5.17) *p* < 0.001*	2.80 (1.57–4.98) *p* < 0.001*	6.60 (3.52–12.38) *p* < 0.001*	6.53 (3.14–13.62) *p* < 0.001*
Child and adult	5.46 (2.72–10.96) *p* < 0.001*	4.20 (1.84–9.61) *p* = 0.001 *	3.47 (1.70–7.11) *p* = 0.001*	1.85 (0.77–4.45) *p* = 0.169	3.24 (1.06–9.84) *p* = 0.039*	3.20 (0.86–11.97) *p* = 0.084

*p < 0.05

^a^Unadjusted odds ratio from univariable logistic regression

^b^Odds ratio from multivariable logistic regression, adjusted for sex, family socio-economic status, teenaged mother, single parent, change in residence, maltreatment, self-control, worry/fearfulness, and adolescent depression

^c^Reference group (OR = 1)

For adult lifetime suicide attempt, unadjusted analysis showed the ‘adult-only’ and ‘child-and-adult’ groups—but not the ‘child-only’ group—to have elevated risk compared to the ‘never-isolated’ group (Table 2). After adjustment for confounders, just the ‘adult-only’ group (OR 2.80, 95% CI 1.57–4.98, *p* < 0.001) retained this higher risk.

For adult recent suicide ideation, the results followed a similar pattern as for lifetime suicide attempt, with higher adjusted risk for just the ‘adult-only’ group (OR 6.53, 95% CI 3.14–13.62, *p* < 0.001) (Table 2).

## Discussion

This paper presents the findings of a longitudinal study of social isolation from a New Zealand birth cohort followed to mid-adulthood. We used four trajectory groups—identified in an earlier study [38]—that mapped well to the onset and persistence of social isolation: never-isolated, child-only, adult-only, and child-and-adult. Our findings add to the literature by showing that time of onset and persistence of social isolation—as represented by four trajectory groups—are differentially related to the presence of the three adult mental health outcomes. This has implications for policy and practice, giving indications as to when during the life course interventions might be targeted to reduce the prevalence of adult depression or suicide attempt/ideation.

Our research question was: Does membership of ‘trajectory’ groups influence adult depression and suicide outcomes? We found that patterns of social isolation in children and adults affected the three adult mental health outcomes with adjusted risks being worse in ‘adult-only’ versus ‘child-only’ groups, worst in the ‘child-and-adult’ group (signifying persistent social isolation) for depression, and worst in the ‘adult-only’ group for suicide attempt/ideation. The risk profiles for the two adult groups (‘adult-only’ and ‘child-and-adult’) were similar. For all three adult outcomes, it appears that the ‘child-only’ group has no greater risk than the ‘never-isolated’ group. The difference in risks between child-onset and persistent groups cannot be explained by more severe symptoms in childhood for the latter group, as symptom levels were very similar in both groups [38].

While we ruled out likely confounding factors, we cannot infer causality from our results. However, given there is evidence that intervening on social isolation can benefit mental health [53], our results suggest that interventions in adulthood may reduce suicide outcomes, while intervening in childhood on those likely to have persistent social isolation [38] may reduce depression. Mechanisms that might explain the link between social isolation and mental health include behavioural, psychological, and physiological pathways [4], and disruptions of the stress–response and increased allostatic load [33]. However, even if the associations reported are not causal, they suggest that social isolation at different life stages is a marker for depression and suicide risk, and so screening for mental health issues among isolated individuals may be warranted.

This study elucidates the relative contribution of social isolation experienced at different stages of the life course and adds weight to evidence on its negative effects on adult mental health. Findings from reviews show that positive aspects of social relationships—including support and extensive networks—have protective effects against depression [40], and that social isolation is strongly associated with suicide outcomes [41, 54]. While concurrent social isolation is associated with depressive symptoms across a range of life stages, the largest effect has been found to occur in adolescence [39]. Our findings indicate that there may be different dynamics for adult depression and suicide outcomes respectively in relation to the timing of social isolation by life stage. Both share the feature that child-only social isolation—unlike adult-only isolation—was not predictive of adult outcomes. However, persistent social isolation (occurring in both childhood and adulthood) was associated with adult depression but not with adult lifetime suicide attempt nor recent suicide ideation (small numbers here). Note that our measure of lifetime suicide attempt was such that attempts may have occurred at any time before the ages of 38 or 45. Longitudinal studies have found that children who experienced social isolation had elevated risks for depression in adulthood [33, 34], and that individuals who had been mostly alone in late childhood were more likely to commit suicide (completed suicide) in mid-adulthood [35]. Child-onset and adult-onset depression—while social isolation may be a common risk factor—have been shown to be related to different child risk factor profiles [55]; this may partly explain our finding that social isolation in childhood was not associated with future adult depression unless social isolation had persisted into adulthood. Aside from social isolation, other concurrent factors such as socio-economic status may also make adults more prone to depression [56] or suicidal ideation or behaviour [57]. It has been found that social isolation may moderate the effect of poor socio-economic status on district suicide rates [42].

In policy and practice terms, understanding the influence of life-course differences in onset and the persistence of social isolation on adult mental health outcomes assists the design and implementation of interventions. Providing early support for families and children is crucial to preventing the onset and to interrupting the persistence of social isolation, and, in turn, reducing the incidence of mental health problems, particularly depression. In the case of lifetime suicide attempt or recent suicide ideation, it appears that adult factors—social isolation and likely other factors not considered here—may play a more influential role, so that intervention efforts may be better concentrated on the adult life stage. As well as the preventative aspect, it has been found that interventions based on reducing social isolation [53], increasing social participation [57, 58], or building social capital [59] may be beneficial for people with existing mental health problems. Further, social group membership has been shown to not only protect against developing depression, but also to reduce and prevent the recurrence of depressive symptoms [60]. However, it is important to recognise that there is no one-size-fits-all solution as people can be socially isolated for different reasons, and thus effective interventions will need to take into account the wider context [37].

There may be a cautionary warning regarding the long-term effects of the COVID-19 pandemic: many children in 2020 and 2021 who are experiencing prolonged social isolation and mental distress during enforced lockdowns [61] may, decades down the track in their adult future, be bearing the harmful consequences in terms of worse mental health. In this future, the prevalence of poor mental health among the population will be greater than the counterfactual (i.e., the hypothetical future where the pandemic had not occurred) and this would have major implications for society as a whole. As well as providing the scaffolding to support individuals and thus prevent social isolation becoming a public health problem, fundamental societal change is needed in the context of social conditions that determine social connection and good mental health, for example, fair socio-economic arrangements, and a value system based on mutual respect and co-operation [62].

### Strengths and limitations

The main strength of this study is the longitudinal information on social isolation measured from childhood to adulthood. This enables not only the analysis of onset but also of any persistence across the life course. We were able to use an existing typology of four trajectory groups—derived from child and adult measures of social isolation.

Limitations involve, first, that our heuristic social isolation measures which were not designed specifically to measure social isolation and only cover one aspect of social isolation, i.e., social disconnectedness [63], and did not assess how the experience was perceived by participants. Social isolation is not the same as, nor highly correlated with, loneliness—also known as perceived social isolation—though the former is a risk factor for the latter [9], both are independently associated with adverse health outcomes, and together may have a synergistic effect [64]. The assessment of both objective social isolation and subjective loneliness are difficult in practice [65] and may make findings difficult to interpret. Our child and adult measures of social isolation were not ideal though both were by way of informant report based on observation of the person and likely to be objective. As in many longitudinal studies, it was not possible to fix past measurement issues. Second, our definition of suicide ideation was broad-brush indicating that any kind was a risk conferred by social isolation, e.g., there was no distinction made between passive and active ideation where the latter suggests greater severity and impact on life outcomes. Third, we assumed that trajectory group and potential confounders preceded adult mental health outcomes, which may not necessarily be the case, though we did control for previous (adolescent) depression in regression analyses. Note that re-analysis without controlling for previous depression did not appreciably change the results, though, for suicide ideation, the estimate for the child-and-adult trajectory increased slightly and became significant (OR 4.07, 95% CI 1.29–12.82, *p* = 0.017). Fourth, we did not account for other formative confounders from early life such as the ability to make and sustain relationships, nor have we accounted for confounding by factors contemporaneous with adulthood, with both issues having potential implications for the form and timing of interventions. Fifth, we cannot infer causality and so cannot rule out reciprocal effects. In particular, the direction of effect regarding lifetime suicide attempt is indeterminate; while information on timing during the life course was available, there were insufficient numbers to analyze suicide attempts after age 38. Finally, the findings derive from analyses of a New Zealand cohort so may not be generalizable to all populations.

### Future research

This paper examines risk factor levels for groupings by social isolation that have an embedded longitudinal component. More sophisticated longitudinal analyses—with the requisite data—offer the possibility of assessing changes in adult mental health outcomes in tandem with changes in social isolation. Sharp inflection points during the life course—perhaps indicating critical periods—could thus be identified where interventions could reasonably be targeted to good effect. Considering later circumstances that might be more relevant to adult outcomes would clarify the effect of social isolation trajectories.

## Conclusion

This study shows that trajectories of social isolation are associated with adult depression, lifetime suicide attempt, and recent suicide ideation. Points of intervention during the life course are suggested by our findings, addressing the onset and persistence of social isolation to improve adult mental health outcomes. Adult depression may be potentially amenable to intervention during either childhood or adulthood, while contemporaneous circumstances seem more relevant for adult suicide outcomes.

## Data Availability

The datasets reported in the current article are not publicly available due to lack of informed consent and ethical approval but are available on request by qualified scientists. Requests require a concept paper describing the purpose of data access, ethical approval at the applicant’s university, and provision for secure data access. All data analysis scripts and results files are available for review.
